# Is body mass index (BMI) or body adiposity index (BAI) a better indicator to estimate body fat and selected cardiometabolic risk factors in adults with intellectual disabilities?

**DOI:** 10.1186/s12872-021-01931-9

**Published:** 2021-03-02

**Authors:** Anna Zwierzchowska, Diana Celebańska, Barbara Rosołek, Krystyna Gawlik, Aleksandra Żebrowska

**Affiliations:** 1 Institute of Sport Sciences, The Jerzy Kukuczka Academy of Physical Education in Katowice, ul. Mikołowska 72a, 40-065 Katowice, Poland; 2 Physiotherapy Department, The Pope John Paul II State School of Higher Education in Biala Podlaska, Sidorska 95/97, 21-500 Biała Podlaska, Poland

**Keywords:** Intellectual disabilities, Body mass index, Body adiposity index, Cardiometabolic risk

## Abstract

**Background:**

The BMI index cannot always be used in people with intellectual disabilities due to neuromuscular coordination disorders and psychological barriers that may hinder conventional body weight measurement. The study aimed to assess the usefulness of BMI and BAI in estimating obesity and body fat in people with intellectual disabilities.

**Methods:**

The first stage of the research involved 161 people with profound intellectual disabilities. Somatic parameters (BM, BH, WC, HC) were measured and BMI, BAI, WHR were calculated. Fifty seven persons with above-normal BMI and BAI were included in the second stage of the study and biochemical parameters were determined (TC, LDL-cholesterol, HDL-cholesterol, TG, GL).

**Results:**

According to both BMI and BAI classifications, most people were overweight or obese. A high correlation of %BF with BMI and BAI indices was observed (r = 0.78). The sensitivity of both indices was 95.65%. In groups with above-normal BMI and BAI, an upward trend was found for mean values of TC, LDL, TG, and GL, with a simultaneous downward trend for HDL. Statistically significant intergroup differences were recorded for TG and GL (*p* < 0.05) for both indices (BMI and BAI).

**Conclusions:**

Our research demonstrated that BAI is complementary to BMI and can be recommended for the estimation of body fat and cardiometabolic risks in people with intellectual disabilities. Due to the ease of measurement, BAI has high utility value.

## Background

Excess body fat (%BF) is a marker of obesity viewed as a chronic and complex metabolic disease that is one of the main risk factors for cardiometabolic diseases, disabilities, and deaths [1, 2]. A reliable and unquestioned estimation of %BF in the human body requires expensive diagnostics in the form of a dual-energy X-ray absorptiometry (DEXA). However, in clinical practice, it is most often conducted in laboratory conditions, thus not being widely used in epidemiological studies. In population studies, body fat is mostly estimated using electrical bioimpedance analysis (BIA), which shows a high correlation with DEXA [3–5]. Despite the increased availability of tools for %BF measurement, professional equipment is not widespread in society, whereas publicly available household scales with body fat analyzers are characterized by large measurement errors [6]. One alternative to complex devices are indices computed based on anthropometric characteristics, which allow for easy and non-invasive estimation of body fat, providing indirect and quick information about the person’s health status.

Body mass index (BMI), considered the obesity index, is a widely used index recommended by WHO for social use, but it is also used in scientific research [7–9]. It defines body weight to height ratio but does not differentiate between muscle and fat mass and its distribution. BMI does not take into account the effect of age, sex, and race, which largely determine the amount of body fat [9–12].

The last decade has seen the verification of the body adiposity index (BAI) developed by Bergman et al. [11]. It has been shown that BAI takes into account both age and sex [13], can be used in both Caucasian and Mediterranean populations [12, 14], and is a sensitive tool in estimating obesity among people with forced sedentary lifestyles [15]. Similarly, the significant sensitivity of the tool was indicated by Godoy-Matos et al. [16] who examined extremely obese women (BAI was more correlated with %BF than BMI). In conclusion, the results of the research to date, although not always unequivocal, reveal both methodological and interpretative limitations of BAI. However, they indicate significantly higher sensitivity of BAI in estimating body fat in obese people than its specificity in population studies.

There are no studies available in the literature on the subject to date that have analyzed indices of obesity and body fat in people with severe intellectual disabilities, [17, 18]. All the more so, the verification of the index in the population of people with intellectual disabilities is important because the measurement of body mass of these people may be flawed due to neuromuscular control disorders and psychological barriers, which lead to problems with the correct load to the tool for measurement of body mass and body composition [19, 20]. At the same time, it has been shown that a severe intellectual disability determines body structure and body fat distribution, which justifies the need to verify the indices of obesity and body fat in the general population [21]. The study aimed to assess the usefulness of the indices of obesity (BMI) and body fat (BAI) in a group of people with intellectual disabilities. It was assumed that BAI is characterized by a higher correlation with body fat (%BF) than BMI and that it is more sensitive in estimating obesity than BMI. Furthermore, based on BMI and BAI, an attempt was made to identify selected cardiometabolic risk factors.

## Materials and methods

**Fig. 1:**
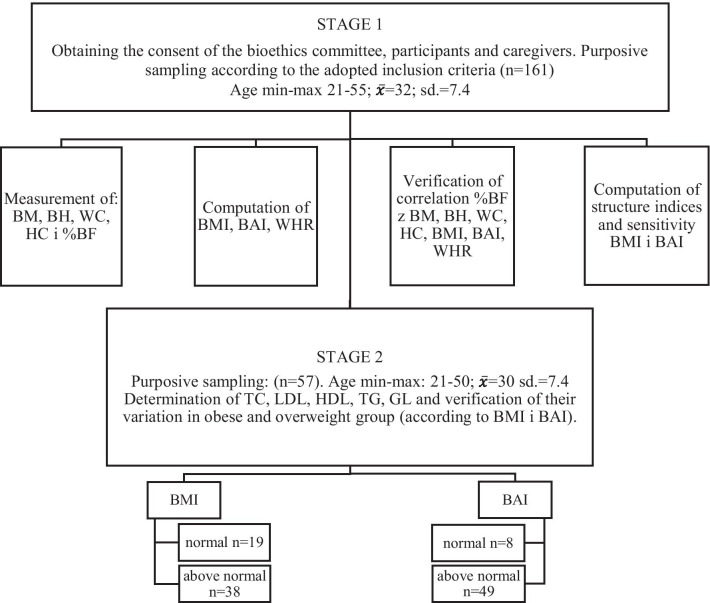
Research methodology

The research was conducted in two stages (Fig. 1), using direct observation, whereas the selection of the respondents was purposive. In the first stage, the inclusion criteria were adopted, i.e. age over 18 years, severe intellectual disability [22], and participation in occupational therapy classes. The aetiology of intellectual disabilities was identified based on health records. It was found that in the case of 75% of the participants, the aetiology was of constitutional origin of the prenatal period, including numerical, sexual and structural chromosomal aberrations (45%). Next, the aetiology related to the diseases suffered by mothers during pregnancy should be indicated (30%), with the focus on exogenous factors, toxic substances, and medicines. The perinatal period, i.e. intrauterine hypoxia, cerebral palsy, improper surgical interventions was associated with 20% cases. The remaining 5% were not classified.

As a result, 161 people (72 women and 89 men) were qualified for the first stage. The following somatic features were measured: body weight (BM), body height (BH), waist circumference (WC), and hip circumference (HC). WC and HC were measured according to the WHO (2011) methodology, whereas %BF was measured using a Tanita MC-780 MA analyzer (Table 1).

**Table 1:** Somatic characteristics of the female and male participants

Sex	Female (n = 72) $\overline{x}$ ± sd	Male (n = 89) $\overline{x}$ ± sd
Age (years)	32.1 ± 8.79	32.0 ± 6.11
BH (cm)	155.8 ± 8.93	171.6 ± 9.48
BM (kg)	66.0 ± 18.70	77 ± 18.55
Waist circumference (cm)	86.2 ± 16.5	92.2 ± 14.7
Hip circumference (cm)	103.5 ± 12.6	102.1 ± 12.6

BMI (related to WHO norms [23]), waist to hip ratio (WHR) (related to WHO norms [24]) and BAI (related to cutoffs as proposed by Bergman et al. [11]) were calculated. The following formula was used for the computation of BAI [11]:

$${\text{BAI}} = \frac{{{\text{hip}}\;{\text{in}}\;{\text{cm}}}}{{{\text{height}}\;{\text{in}}\;{\text{m}}}} - 18$$

In the second stage of the study, persons who did not express the consent for blood sampling were excluded from the study. As a result, the lipid profile (total cholesterol (TC), LDL-cholesterol, HDL-cholesterol, and triglycerides (TG)) and glucose (GL) concentration were evaluated in 57 people with intellectual disabilities. The blood samples were obtained on an empty stomach in the morning (the participants were examined at least 12 h after their last meal). The determination of biochemical parameters was carried out with the use of Randox diagnostic tests in the analytical laboratory of the Jerzy Kukuczka Academy of Physical Education in Katowice.

The research was part of the project "Lifestyles and the threat of the diseases of affluence in adults with disabilities" conducted by the Department of Physical Education and Adapted Physical Activity of the Jerzy Kukuczka Academy of Physical Education in Katowice.

The research project received a positive opinion of the Bioethics Committee of the Academy of Physical Education in Katowice (Resolution of 8 March 2012 No. 9/2012). The study protocol conforms to the ethical guidelines of the 1975 Declaration of Helsinki as reflected in a priori approval by the institution's human research committee. The subjects were informed about the purpose and procedure of the study, expressed their written informed consent for the participation, and were allowed to withdraw from the participation at any stage of the study. Written consents for the incapacitated individuals were obtained from legal guardians. The data obtained in the research were secured in accordance with the Personal Data Protection Act of 10 May 2018 (Journal of Laws of 2018, item 1000).

### Statistical analysis

The normality of distribution was evaluated for age and somatic features (BM, BH, WC, HC), indices (BMI, BAI, WHR, %BF), and parameters (TC, HDL, LDL, GL, TG) (Kolmogorov–Smirnov test) (n = 161). The correlation of %BF with features and indices (Spearman’s rank correlation) was verified. The most correlating indices were selected for further analysis (BMI, BAI). In accordance with the norms (BMI) (group with normal values (18.5 < BMI < 24.9) and excess body mass of (BMI ≥ 25.0)) and cutoffs (BAI) (cutoffs by age and gender rates for women > 35% and for men > 22%), the subjects were divided into groups and, based on them, the significance of differences between the structure indices for groups was calculated and verified.

The sensitivity of BMI and BAI was verified relative to %BF. The statistical significance of differentiation of mean values of biochemical parameters (GL, TC, HDL, LDL, TG) between groups of patients with normal and above-normal BMI and BAI cutoff points (U test) (n = 57) was verified. The statistical significance of differences was set at *p* < 0.05.

## Results

The presence of correlations between %BF and somatic features was verified and the strongest statistically significant correlation was found between %BF and BMI and between %BF and BAI (Table 2). Therefore, further analysis was conducted based on these two indices.

**Table 2:** Relationship of somatic features and indices relative to body fat

	BM	BH	WC	HC	BMI	BAI	WHR
%BF							
r	0.53	− 0.26	0.63	0.77	0.78	0.78	0.25
p	0.001	0.001	0.001	0.001	0.001	0.001	0.001

**Fig. 2:**
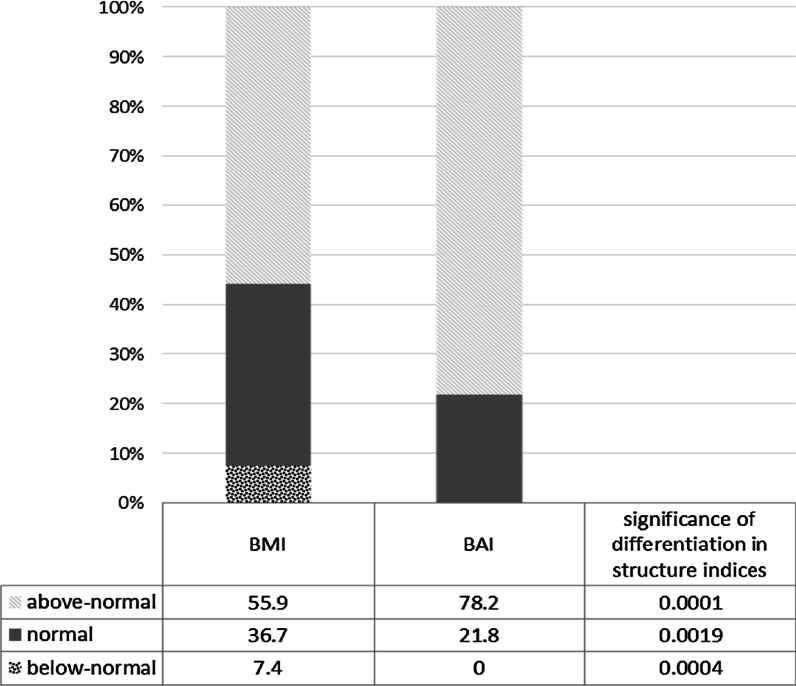
Group structure according to BMI norms and BAI cutoff points (n = 161)

After the classification of the participants according to norms and BAI cutoff points for women and men, it was shown that the vast majority of them were obese or overweight, regardless of the classification criterion (BMI/BAI). The calculated structure index revealed statistically significant differences between groups (BMI n = 90; 55.9% and BAI n = 126; 78.3% *p* < 0.01). Furthermore, assuming the %BF as a golden standard, the sensitivity of BMI and BAI indices was verified and it amounted to 95.65% for both indicators (Fig. 2).

In the second stage, mean values of biochemical parameters were compared in groups classified according to BMI norms and BAI cutoff points (Table 3). The expected upward trend of mean values of TC, LDL, TG, GL was observed, with a simultaneous downward trend for HDL both in the group with above-normal BMI and in the group with BAI above cut-off points. Statistically significant intergroup differences were recorded for TG and GL (*p* < 0.05) for both indices (BMI and BAI) (Table 3).

**Table 3:** Mean values of biochemical parameters according to BMI norms and BAI cutoff points

	BMI			BAI		
	Normal	Above-normal	*p* value	Normal	Above-normal	*p* value
	n = 19	n = 38		n = 8	n = 49	
TC (mg/dl)	156.2 ± 28.9	172.9 ± 35.8	–	154.6 ± 29.6	169.4 ± 34.9	–
HDL (mg/dl)	65.1 ± 18.8	58.9 ± 20.6	–	70.7 ± 22.1	59.3 ± 19.5	–
LDL (mg/dl)	76.1 ± 28.0	88.8 ± 32.0	–	70.2 ± 28.8	86.9 ± 31.1	–
TG (mg/dl)	75.0 ± 26.3	128.8 ± 70.2	0.0004	68.0 ± 16.9	117.9 ± 66.5	0.0085
GL (mg/dl)	84.0 ± 7.5	92.1 ± 15.8	0.0486	82.7 ± 5.7	90.5 ± 14.7	0.0429

## Discussion

Obesity has been identified in a substantial part of the population of people with intellectual disabilities, leading to increased cardiometabolic risks [25]. This leads to the search for reliable, cheap, and easy to use tools to estimate obesity and body fat in this group of disabilities. It has been shown that %BF measured using the bioimpedance method has a high correlation with BMI, i.e. the index for estimating obesity that is most frequently recommended for the general population [14, 26, 27]. These findings were consistent with our results as both BMI and BAI, also verified using the bioimpedance (BIA) method, showed a statistically significant correlation (between BMI and %BF (r = 0.78) and between BAI and %BF (r = 0.78) *p* < 0.01). The results obtained suggest that both BMI and BAI may be a recommended tool for estimation of excess weight of patients with intellectual disabilities.

It should be emphasized that few studies have assessed the use of BMI in the population of people with intellectual disabilities and often involved groups that differ in terms of the aetiology, age, comorbid conditions, ways of interpreting the data (growth charts or norms for estimation of obesity). Consequently, the results of these studies are ambiguous in terms of the validity of the use of BMI in this group of people with disabilities [4, 18, 28]. The BAI index, which has been popular in recent years in estimating body fat, is gaining increasing interest. The authors of the index point to significant links between BAI and the estimation of cardiometabolic disease risk, which increases its potential for the interpretation of body fat [11]. However, the results of the research conducted to date in populations of different ethnicity, age, gender, disability, and metabolic disease syndromes, are varied, without finally confirming its diagnostic importance [12, 14–16, 29–31].

However, studies to date on the BAI index among people with intellectual disabilities indicate its tendency to overestimate body fat [18, 32, 33], which would suggest its low specificity for this population. Our findings are consistent with this thesis because the sensitivity of the test for the analyzed group for both BAI relative to %BF and BMI relative to %BF was at the level of 95%, although the group structure based on the BAI cutoff point and BMI norms was significantly different (*p* < 0.01). Only a detailed analysis of the second stage of the study demonstrated that people classified outside the BAI cutoff points often remained in the normal range according to BMI classification. As expected, the values of biochemical parameters were higher both in the group of people with above-normal BMI and people with BAI above cutoff points. Furthermore, it was observed that the values of biochemical parameters of lipid profile indicated obesity and cardiometabolic risks, revealing statistically significant intragroup differentiation. Both in the group analyzed according to BMI norm and BAI cutoff points, there were statistically significant differences for TG and GL [BMI (TG = 75.0 ± 26.3/128.8 ± 70.2) and (GL = 84.0 ± 7.5/92.1 ± 15.8)]; [BAI (TG = 68.0 ± 16.9/117.9 ± 66.5) and (GL = 82.7 ± 5.7/90.5 ± 14.7)] at *p* < 0.01. No statistically significant differences for other biochemical parameters (HDL and LDL) were demonstrated, which suggests that both BMI and BAI are sensitive in estimating cardiometabolic risk only for TG and GL. Our results are consistent with the theses presented by Lizak et al. [13], Zwierzchowska et al. [15] concerning higher sensitivity and low specificity of BAI in estimating cardiometabolic risks in groups of people with intellectual disabilities.

### Strengths and weaknesses of the study

In conclusion, the small number of studies on this problem and, consequently, the lack of comparability with our research is a limitation and weakness of this study. However, the fact that similar sensitivity of BAI and BMI was demonstrated unequivocally and that BAI index differentiates the group in terms of biochemical parameters identifying cardiometabolic syndrome allows for the recommendation of BAI as complementary to the BMI index. At the same time, it should be noted that only anthropometric features related to the length and width (BH and WC) are taken into account in estimating BAI, which can sometimes make it easier to use. It seems that our results provide the answer to the study by Jaffrin [6], who indicated a measurement error in body weight and composition. This situation may be associated with frequent neuromuscular control disorders and psychological problems of people with intellectual disabilities, and, consequently, with an accurate estimation of BMI.

We observed a strength of the present study in this respect because we showed a prognostic value of BAI similar to BMI and its utilitarian potential, which can lead to the optimization of diagnostics for people with intellectual disabilities.

## Conclusions

1. The BAI index shows good sensitivity but low specificity for estimating body fat among adults with intellectual disabilities. Furthermore, the BAI index is recommended as complementary to BMI in the prediction of cardiometabolic disease risks.
2. The BAI index has a high utility value due to the ease of collecting data used to calculate it, which is particularly important in the case of comorbid intellectual and motor disabilities (difficulty in maintaining a habitual body posture).

## Data Availability

The datasets used and/or analysed during the current study are available from the corresponding author on reasonable request.

## Abbreviations

TC: Total cholesterol
LDL-cholesterol: Low density lipoprotein
HDL-cholesterol: High density lipoprotein
TG: Triglycerides
GL: Glucose
BMI: Body mass index
BAI: Body adiposity index
WHR: Waist hip ratio
WC: Waist circumference
BM: Body weight
BH: Body height
HC: Hip circumference
%BF: Body fat percentage
WHO: World Health Organization
DEXA: Dual-energy X-ray absorptiometry
